# How to locate the dural defect in a spinal extradural meningeal cyst: a literature review

**DOI:** 10.1186/s41016-022-00291-3

**Published:** 2022-08-31

**Authors:** Qiang Jian, Zhenlei Liu, Wanru Duan, Fengzeng Jian, Zan Chen

**Affiliations:** Department of Neurosurgery, Xuanwu Hospital, Capital Medical University, Beijing, 100053 China

**Keywords:** Spinal extradural meningeal cyst, Dural cyst, Dural defect, Communication, Fistula

## Abstract

Spinal extradural meningeal cysts (SEMCs) are rare lesions of the spinal canal. Although closure of the dural defect can achieve satisfactory therapeutic effects, locating the fistula is difficult. This review summarizes the methods for locating the fistula of SEMCs and the distribution and features of fistula sites.

This was a non-systematic literature review of studies on SEMCs. We searched PubMed for English-language articles to summarize the methods of locating the defect. The search words were “epidural arachnoid cyst,” “dural cyst,” “epidural cyst,” and “epidural meningeal cyst.” For the defect location component of the study, case reports, studies with a sample size less than four, controversial ventral dural dissection(s), and undocumented fistula location reports were excluded.

Our review showed that radiography and computed tomography (CT) may show changes in the bony structure of the spine, with the largest segment of change indicating the fistula site. Occasionally, magnetic resonance imaging (MRI) can show a cerebrospinal fluid (CSF) flow void at the fistula site. The middle segment of the cyst on sagittal MRI, the largest cyst area, and cyst laterality in the axial view indicate the fistula location. Myelography can show the fistula location in the area of the enhanced cyst and subarachnoid stenosis. Digital subtraction or delayed CT can be used to observe the location of the initial cyst filling. Cine MRI and time-spatial labeling inversion pulse techniques can be used to observe CSF flow. Steady-state image construction interference sequence MRI has a high spatial resolution. Neuroendoscopy, MRI myelography, and ultrasound fistula detection can be performed intraoperatively. Moreover, the fistula was located most often in the T12–L1 segment.

Identifying the fistula location is difficult and requires a combination of multiple examinations and experience for comprehensive judgment.

## Background

**Fig. 1:**
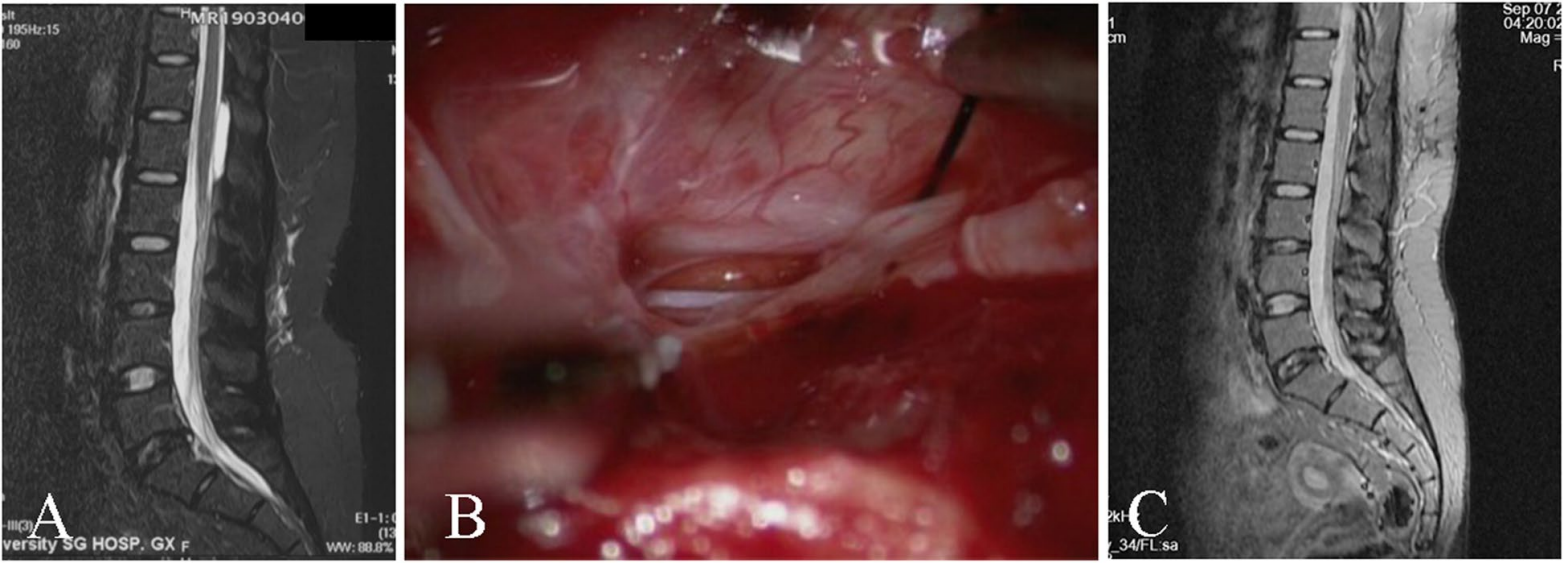
A 26-year-old woman with lower back and left buttock pain for 10 years, aggravating for 1 year. Preoperative MRI showed a SEMC. The dural defect was sutured intraoperatively. One-year follow-up imaging showed no recurrence of the cyst

**Fig. 2:**
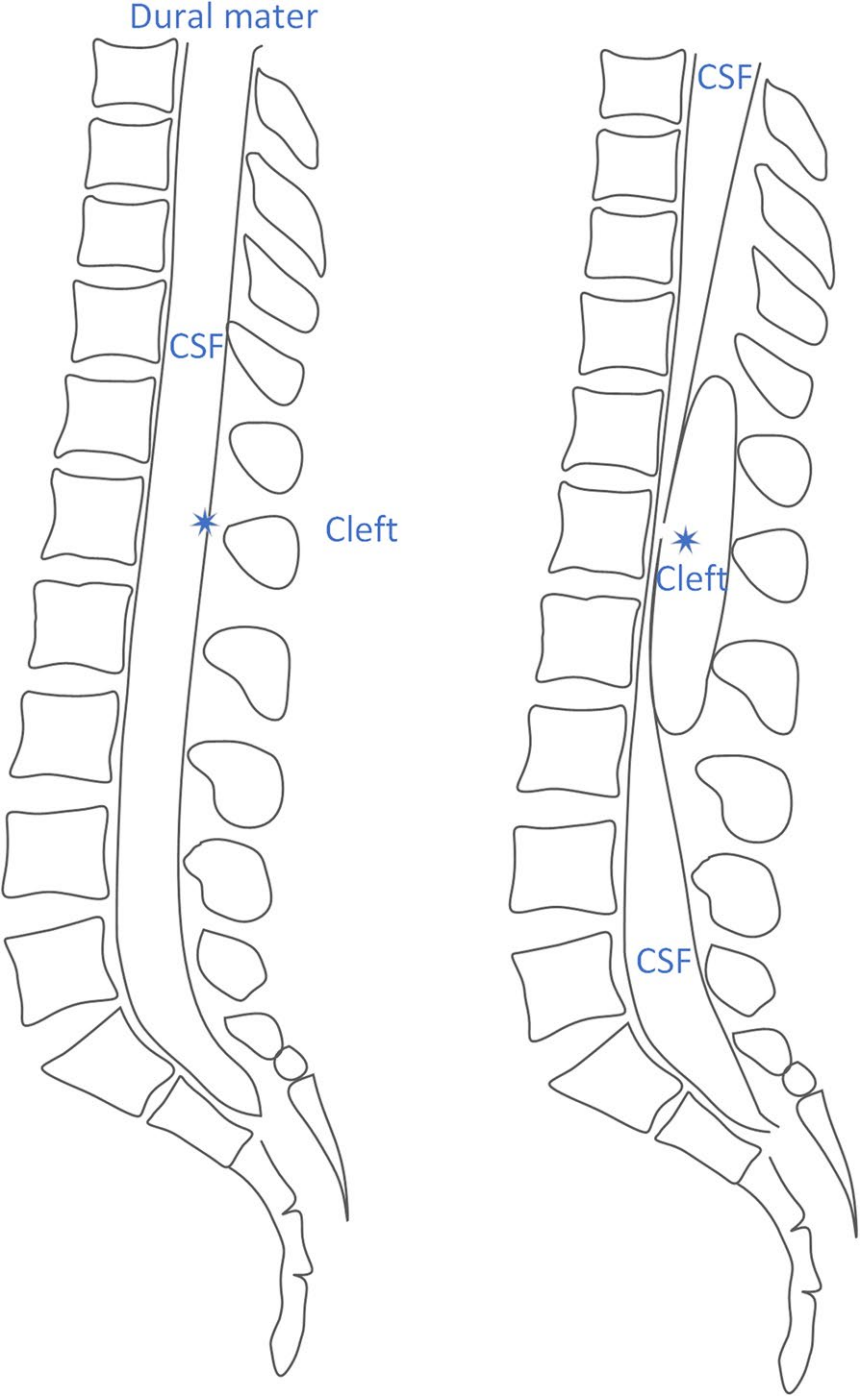
The widely accepted theory of pathogenesis is that the subarachnoid space and cyst communicate through a dural defect

Spinal extradural meningeal cysts (SEMCs) are rare lesions in the spinal canal and account for approximately 1% of epidural lesions [1]. Their etiology and pathogenesis are controversial. Although noncommunicative cysts have been reported, presently, the widely accepted theory of pathogenesis is that the subarachnoid space and cyst communicate through a dural cleft (Fig. 1), which may arise from congenital dural developmental deficits or acquired trauma [1–3]. Under the influence of cerebrospinal fluid (CSF), the arachnoid membrane herniates from the fistula and forms an extradural cyst (Fig. 2). Possible reasons for cyst enlargement include the secretory function of the inner cells of the cyst, the osmotic pressure gradient between the cyst and the subarachnoid space, CSF pulsation, and a one-way valve effect of the fistula [1, 4–9]. Surgical treatment includes total cystectomy, partial excision of the cyst wall, fistula closure, cyst fenestration, and shunting of the cyst [10, 11]. However, because such cysts communicate with the subarachnoid space, most researchers believe that cleft closure is the most effective measure, and cystectomy is unnecessary [4, 12, 13]. Therefore, identifying the location of the fistula preoperatively is the key to performing minimally invasive surgery and treating this type of cyst. This literature review summarizes the techniques for locating the fistula, and the characteristics and distributions of fistula sites, to guide surgical treatment (Table 1).

**Table 1:** Review of the localization method of spinal extradural meningeal cysts

Localization method	Signs	Authors
Spinal routine examination	Plain radiography: obvious enlargement of intervertebral foramina and interpedicle distance	Lee et al., Xu et al.
	CT: severe scalloping changes on the posterior edge of the vertebral body	Lee et al., Xu et al.
	MRI: flow void, dominant cyst laterality in axial view, segment with the largest cyst area in axial MRI, middle segment of the cyst in sagittal MRI	Lee et al., Xu et al., Özdemir et al., Lee et al., Paredes et al.
None real-time contrast examination	Myelography: narrow enhancement area between the subarachnoid space and the cyst	Congia et al., Neo et al.
	CT myelography: narrow enhancement area between the subarachnoid space and the cyst, site where the contrast agent first fills the cyst	Lee et al., Ball et al., Funao et al., Liu et al., DiSclafani et al., Morizane et al., Tanaka et al.
	MRI myelography: flow void	Miyamoto et al.
Digital subtraction contrast examination	Digital subtraction cystography: site where the contrast agent first fills the subarachnoid space from the cyst; verify and determine the laterality in the frontal view	Gu et al.
	Two-needle puncture digital subtraction myelography technology: site where the contrast agent first fills the cyst	Ying et al.
	Digital subtraction myelography: site where the contrast agent first fills the cyst	Lee et al.
Special MRI	Cine MRI: flow void	Neo et al., Funao et al., Morizane et al.
	Steady-state image construction interference sequence (CISS) MRI: communication between the subarachnoid space and the cyst	Nakagawa et al.
	Time-spatial labeling inversion pulse (T-SLIP) MRI: hyperintense signal CSF flow from the subarachnoid space into a hypointense signal cyst	Ishibe et al.
Intraoperative method	Neuroendoscopy: endoscopic findings revealed a dural fistula	Funao et al., Ying et al.
	Intraoperative ultrasound: pulsating cerebrospinal fluid inflow; inner wall of the cyst continued to swell and collapse	Kanetaka et al., Morizane et al.
	Intraoperative sequential dynamic MRI myelography: site with irregular multilobulated morphology	Mishra et al.

## Preoperative examination

### Routine spinal examination: plain radiography, computed tomography (CT), and magnetic resonance imaging (MRI)

#### Plain spinal radiography and CT

**Fig. 3:**
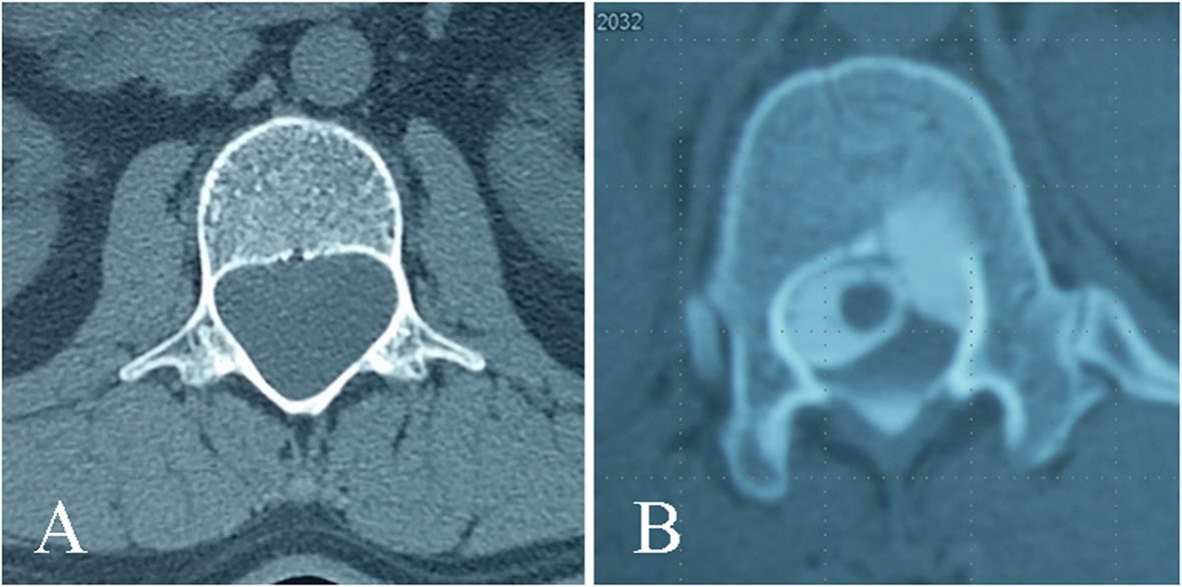
Several indirect signs on CT findings. **A** Axial CT revealed the widening of interpedicle distance and thinning of the pedicle. **B** Axial CT cystography revealed bony scalloping erosion of the posterior edge of the vertebra. Although both the subarachnoid space and the cyst were developed, no cleft was found

Since the cyst in the spinal canal tends to continuously expand, the hydrostatic pressure on the surrounding structures, arising from this expansion, may cause changes in the spine. Radiography may reveal widening of the intervertebral foramen and interpedicle distance, thinning of the pedicle, and even kyphosis deformity. CT may show more subtle changes, such as thinning of the lamina and bony scalloping erosion of the posterior edge of the vertebra [14–16] (Fig. 3).

Lee et al. [13] and Xu et al. [17] analyzed the diagnosis and treatment of eight and ten cases of SEMCs, respectively. Severe scalloping changes on the posterior edge of the vertebral body on CT were considered positive and suspected signs of defect location in their study. Additionally, the level of the most obvious enlargement of the intervertebral foramina and interpedicle distance shown on radiography suggested the segment of the fistula in the study by Xu et al. [17].

#### Spinal MRI

**Fig. 4:**
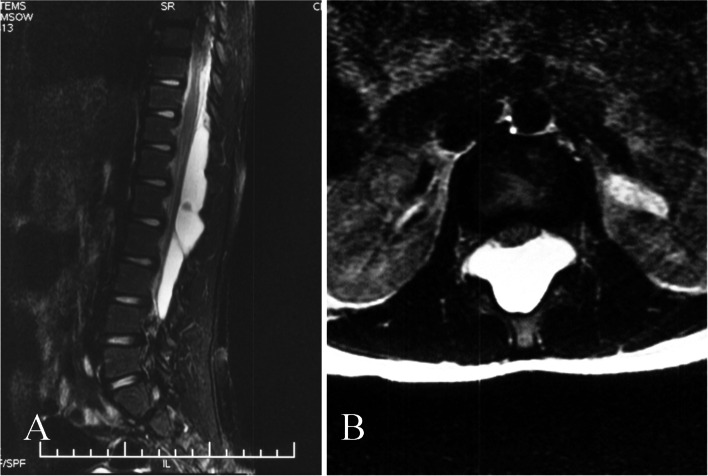
Flow void on MRI. A 7-year-old boy had weakness of the lower extremities, with dysuria, for 3 days. **A** Preoperative sagittal MRI showed a flow void with a SEMC at L1 level. **B** No flow void was found in axial MRI

As a noninvasive examination is the first choice in the diagnosis of SEMCs, MRI is useful for determining the exact location and span of the lesion, as well as the relationship of the lesion with the spinal cord. Enhanced MRI is used to identify other cystic lesions [16]. Furthermore, CSF occasionally flows rapidly through the fistula, between the cyst and the subarachnoid space. Thus, the MRI signal cannot be detected as it presents a low signal in either T1-weighted or T2-weighted images, showing a flow void effect [17] (Fig. 4).

Flow voids in T2-weighted MRI images were considered positive and suspected signs of defect location in the studies by Lee et al. [13] and Xu et al. [17]. Xu et al. [17] additionally proposed that a cyst with a dominant laterality on axial MRI also indicated laterality of the fistula. Lee et al. [18] proposed that the segment with the largest cyst area on axial MRI, and the middle segment of the cyst on sagittal MRI, may represent the level of the fistula.

These routine spinal examinations (plain radiography, CT, and MRI) can only suggest the location of the fistula through indirect signs, but such indirect signs can easily be missed. MRI is a necessary examination for the diagnosis of SEMCs. Although many researchers have reported success with flow voids in predicting fistula levels [19, 20], the sign does not always occur. Radiograph and CT findings of indirect bony signs should therefore be carefully reviewed when the examination methods are limited.

### Non-real-time contrast examination: myelography, CT myelography, and MRI myelography

Myelography refers to the injection of contrast agent into the spinal subarachnoid space, after which scanning can be performed with plain radiography (myelography), CT (CT myelography), or MRI (MRI myelography) to reveal the dura and spinal cord [21].

#### Myelography

Congia et al. [22] diagnosed one case of thoracic paravertebral SEMC through anteroposterior myelography. The SEMC communicated with the subarachnoid space through the intervertebral foramina.

#### CT myelography

Lee et al. [13] proposed that CT myelography could reveal the enhancement of the subarachnoid space and cyst, and the narrow enhancement area between them suggested the neck of the cyst, formed at the site of the dural defect. Ball et al. [23] reported the use of dynamic CT myelography to display the subarachnoid space and cyst fistula in patients with ventral epidural meningeal cysts. In the prone position, the contrast agent was injected into the intrathecal space, and the patient’s hip was elevated. CT was used to monitor the movement of the contrast agent from the caudal to the cranial region. The fistula was located at the site where the contrast agent first filled the cyst. In their study, the fistula was successfully identified in three (3/4) patients. Funao et al. [12] performed myelography and CT myelography to identify a fistula in 12 patients with SEMCs, and the fistula was identified in seven patients. However, Morizane et al. [24] performed CT myelography in 12 patients, and fistulas were found in only two patients.

The entrance of contrast agent into the cyst may be prolonged due to the presence of a narrow fistula. Delayed CT is therefore helpful in locating the communication between the cyst and subarachnoid space. However, researchers have reported different strategies regarding the timing of scanning after contrast injection. Tanaka et al. [25] performed a CT scan after intrathecal injection of a contrast agent, immediately, 10 min later, and 1 h later. Liu et al. [16] proposed that CT myelography is crucial for displaying the site of the fistula and should be performed immediately after the injection, 3 h later, and the next morning. A greater amount of contrast agent fills the cysts in delayed-phase CT for a better display. Disclafani et al. [14] advocated a CT scan 8 h after injection for improved observation of the fistula position.

#### MRI myelography

Miyamoto et al. [26] reported a case in which MRI myelography revealed the location of the fistula in a SEMC. Cine MRI failed to locate the fistula preoperatively. Thus, MRI myelography was performed, and coronal reconstruction revealed a low-signal flow void between the subarachnoid space and the cyst on the right side at the T12 level. The location of the fistula was confirmed intraoperatively.

**Fig. 5:**
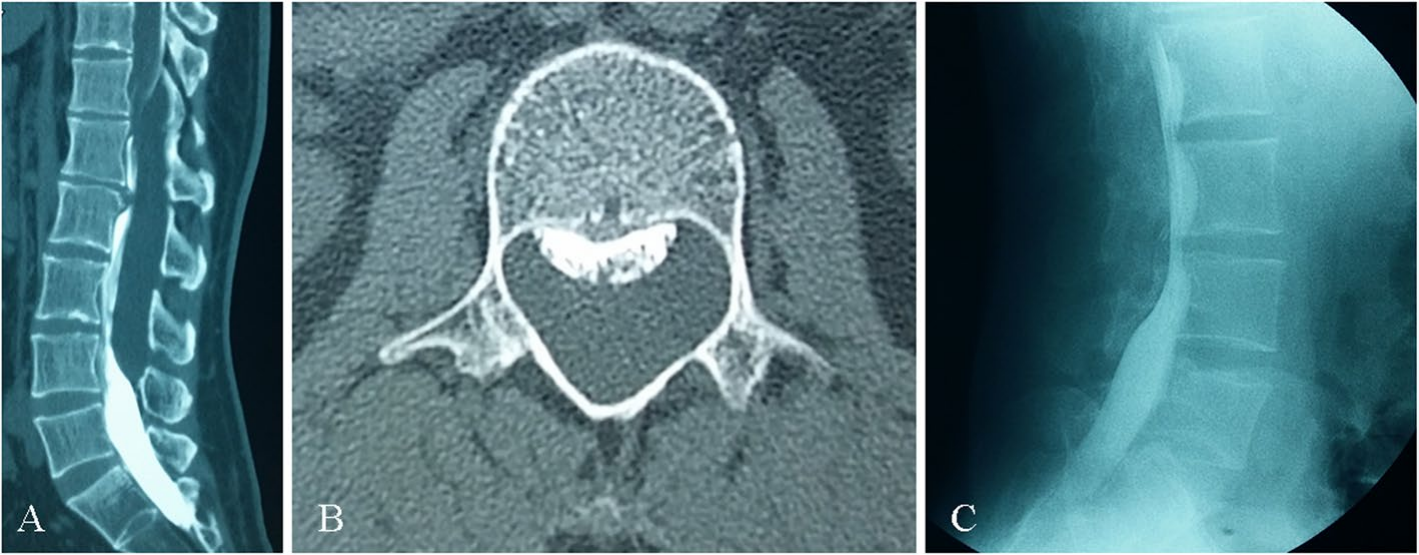
Myelography. The difficulty in performing myelography is the timing of the scan after injection. **A**, **B**, and **C** The cyst did not even develop at the time of scan

Myelography is a common examination to locate fistulas. Although these researchers reported successful results, diagnoses are still missed in this type of examination. Plain myelography may reveal cyst development. However, because of the overlap of the cyst with the subarachnoid space in the plain radiography image, the underlying cyst pedicle may not be observed. CT myelography is considered the first choice for preoperative exploration of the fistula [16], but it is difficult to determine the timing of the scan, and it often fails to show the location of the earliest entry of the contrast agent into the cyst (Fig. 5). Multiple scans may increase the risk of radiation exposure in patients. MRI myelography is considered a useful method for detection of fistulas after failure of cine MRI [26], but it has been less frequently reported.

### Real-time digital subtraction contrast examination: digital subtraction cystography or myelography

Although there is abnormal CSF flow in patients with SEMCs, this flow is difficult to detect on static examinations such as conventional MRI and CT. Contrast examination, which is expected to show communication between the cyst and subarachnoid space, is especially important with regard to the timing of the scan, which is otherwise likely to fail. Rimmelin et al. [27] performed myelography and cystography on two patients. In both cases, the cysts were noted with the contrast media, but no fistulas were found. In one case, the cysts were observed 5 h after the injection. The researchers suggested that the narrow cyst pedicle made it difficult for the contrast agent to pass through, and delayed CT was needed for the cyst to be observed. Even if the cyst was noted with contrast media, the signal of the cyst can still be the same as that of the subarachnoid space, making it difficult to identify the fistula location [28]. Therefore, some researchers have adopted real-time digital subtraction contrast examination.

#### Digital subtraction cystography

Gu et al. [29] found fistulas in six patients using digital subtraction cystography. First, with the patient prone, the cyst was punctured using a 12-cm spinal needle through the posterior approach, under fluoroscopy, after sterile preparation and local anesthesia. After the puncture needle was confirmed to lie in the cyst under fluoroscopy, 10 mL of the fluid was aspirated from the cyst to relieve pressure. The patient was instructed to hold his/her breath while being injected with 3–4 mL of contrast medium, and digital subtraction was performed in the lateral projection at a rate of one frame per second. A clear flow of contrast agent from the cyst entering the subarachnoid space was identified as a fistula. The frontal view was used to verify and ascertain the laterality of the fistula. According to the frontal image, the location of the fistula was divided into pedicle, infrapedicle, and disc levels. In these six patients, no fistula was found on preoperative CT myelography and MRI, but digital subtraction cystography was able to locate the fistulas.

#### Two-needle puncture digital subtraction myelography technology

Ying et al. [30] reported a successful case of fistula location with real-time contrast imaging using a double-needle puncture under fluoroscopy. First, cystography was performed. A CT scan 1 h later did not reveal subarachnoid space development. Moreover, MRI did not reveal cyst shrinkage after approximately 20 mL of fluid was extracted from the cyst. They considered the existence of a one-way valve mechanism in the fistula that enabled fluid from the subarachnoid space to pass into the cyst. Subsequently, a two-needle puncture fluoroscopic real-time myelography technique was developed. Under fluoroscopy, two needles were inserted into the cyst and the L3/4 subarachnoid space, respectively, to drain the fluid. Thus, the pressure between the two spaces was balanced. Thereafter, 10 mL of contrast medium was injected into the subarachnoid space. Under fluoroscopy, the contrast agent was observed to enter the cyst at the T12/L1 level. A subsequent high-resolution CT scan also revealed a left fistula.

#### Digital subtraction myelography

Lee et al. [18] performed digital subtraction cystography, digital subtraction myelography, and CT in one patient. No fistula was found on real-time cystography imaging, and follow-up CT showed that the contrast agent had reached the subarachnoid space. Although cyst development was observed with digital subtraction myelography performed thereafter, no clear fistula was found with the contrast agent. Finally, they comprehensively inferred the site of the fistula based on three factors: (1) in digital subtraction myelography, the region where the contrast agent first filled the cyst was T12–L1; (2) in sagittal MRI, the middle of the cyst was L1; and (3) in axial MRI, the segment with the largest cyst area was T12–L1. These three factors all suggested that the fistula was located at the T12–L1 level, and laminectomy and fistula repair were successfully performed at T12.

**Fig. 6:**
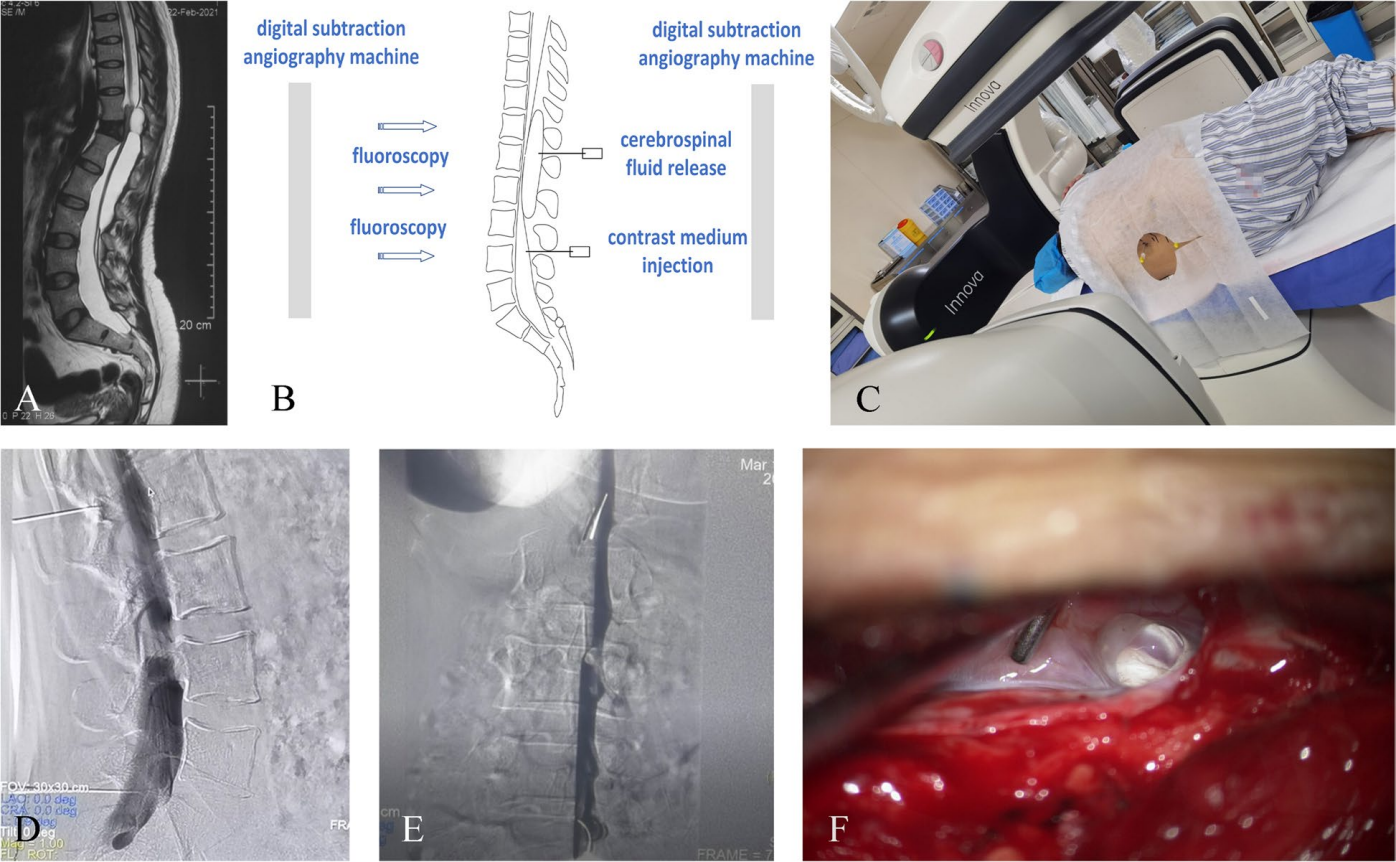
Two-needle puncture digital subtraction myelography technology. A 35-year-old woman had low back pain for 4 years. **A** Preoperative MRI revealed a butterfly vertebra and a SEMC. **B** and **C** We performed two-needle puncture digital subtraction myelography which failed. **D** and **E** No cleft was found during digital subtraction myelography. **F** The cleft was found during intraoperative exploration. The drawback of these digital subtraction contrast examination techniques is that the boundary of the subarachnoid space and cyst cannot be shown

Preoperative real-time myelography or cystography is a new method used to detect fistulas. It is simple to operate and easy to obtain. In contrast to traditional imaging methods, digital subtraction myelography or cystography is characterized in real time, and the direction of the contrast medium flow can be observed in real time under fluoroscopy. However, the drawbacks of these techniques are also apparent. In contrast to MRI, the visualization of the boundary between the cyst and subarachnoid space is unclear. When the contrast agent reaches the fistula through the subarachnoid space, the overlapping effect of fluoroscopy between the cyst and subarachnoid space makes it difficult to determine the location where the contrast agent first fills the cyst (Fig. 6).

### Special MRI imaging

The advantage of MRI is the visualization of soft tissue, which can clearly show the boundary between cyst and subarachnoid space [16]. Some researchers have noted its advantages and imaged with special MRI techniques, which can directly boost the visualization of the dynamic flow of CSF.

#### Dynamic MRI

Dynamic MRI can demonstrate the movement of fluid and nerve tissue, thus showing the site of the dural defect and location of the pulsating turbulent flow void. Neo et al. [31] presented a case of SEMC diagnosed with true fast imaging, with a steady-state precession technique to perform cine MRI, which successfully located the fistula site. A flow void on the left side of the L1 level was considered the fistula site. Subsequent posteroanterior myelography revealed the intervertebral foramen at L1–L2. Myelography confirmed the discovery by cine MRI. Morizane et al. [24] performed cine MRI in six patients, fistulas were found in four of these patients, and all the fistulas were located at the T12–L1 segment. However, Funao et al. [12] reported that only two of 12 cases showed fistulas on cine MRI.

#### Steady-state image construction interference sequence (CISS) MRI

Nakagawa et al. [32] reported a case of a large SEMC and the advantage of CISS MRI. Preoperative CT, MRI, cine MRI, and intraoperative neuroendoscopy did not reveal a fistula. Partial excision of the cyst was performed to relieve symptoms. Postoperative three-dimensional (3D) CISS MRI revealed the site of the dural defect at the S1 level. The fistula was sutured in the second operation. Cine MRI has a high time resolution but cannot achieve high spatial resolution [33]. In cases of subarachnoid obstruction with poor CSF flow, defects may not be detected with cine MRI. However, 3D CISS MRI is an MRI technique used to visualize the fine nerve and vessel structure in CSF using a high spatial resolution [34, 35]. This case demonstrates the unique advantage of 3D CISS MRI for this type of large cyst with a small fistula.

#### Time-spatial labeling inversion pulse (T-SLIP) MRI

Time-spatial labeling inversion pulse (T-SLIP) MRI, a type of cine MRI, can visualize fluid flow by inversion pulse without invasion [36–38]. Ishibe et al. [28, 39] applied the T-SLIP technique to invert the CSF signal in the cyst (hypointense signal), while the signal in the subarachnoid space remained unchanged (hyperintense signal). Thus, CSF flow into the cyst from the subarachnoid space (hyperintense signal) could be detected. CSF flow can be further classified into intermittent and persistent flows. Three (3/3) patients with SEMCs were successfully diagnosed in their study.

Special MRI techniques have been reported in recent years. Cine MRI can be used for dynamic observation [24], CISS MRI has a higher spatial resolution [32], and T-slip MRI can visualize flow signals more clearly [28, 39]. Therefore, special MRI techniques are additional methods for detecting fistulas. However, such techniques may be difficult to perform in non-tertiary medical institutions and may require multi-department collaboration. Because of its noninvasive nature, special MRI techniques have a promising future after its popularization.

## Intraoperative detection methods

### Neuroendoscopy

Funao et al. [12] and Ying et al. [30] proposed the application of neuroendoscopy to detect fistulas in cysts. After the fistula was identified with neuroendoscopy, the fistula was sutured using a microscope. Funao et al. performed a small fenestration on the dorsal wall of the cyst and placed a 3.0-mm-diameter flexible neuroendoscope into the cyst. The flexible endoscope could precisely identify the fistula. However, in the multilevel and multiloculated cases reported by Nakagawa et al. [32], endoscopic exploration was not successful.

### Intraoperative ultrasound

Kanetaka et al. [40] reported a successful case of fistula detection using intraoperative ultrasound. Preoperative MRI showed the SEMC at T11–L3, but CT myelography and phase-contrast MRI did not reveal the fistula. After T11–L3 laminectomy, Doppler ultrasound was performed using a water-path imaging technique. Doppler ultrasonography revealed pulsating CSF inflow on the left side of L1, as well as a valvular structure near the fistula. Morizane et al. [24] performed an ultrasound examination of the cyst after intraoperative laminectomy in two patients and found that the inner wall of the cyst continued to swell and collapse, indicating that the nerve root acted as a single valve mechanism at the fistula.

### Intraoperative sequential dynamic MRI myelography

Mishra et al. [41] reported a new technique for the detection of fistula sites using intraoperative sequential dynamic MRI myelography. Preoperative MRI revealed a large cyst extending from T10 to S1. CT myelography is difficult to perform because of the cyst-occupying effect and lack of puncture space in the L3/4 segment. Contrast media injected through the L3/4 space may not be able to enter the subarachnoid space to indicate the location of the fistula. Thus, they first removed the T8 lamina above the cyst and implanted a catheter in the subarachnoid space. Subsequently, the patient was transferred to an MRI machine under general anesthesia. After the catheter position was determined to be satisfactory, the contrast agent was injected from the catheter, and scanning was performed four times until the cyst was fully developed. The location with an irregular multilobulated morphology on the left side of L1 was determined to be the fistula site.

Neuroendoscopy can achieve success in exploring the fistula in the cyst cavity as it has limited invasiveness, but some single cysts have multilocular compartments, making fistula exploration difficult. Second, for long-segment cysts, there may be inevitable omissions using neuroendoscopy. Ultrasonography requires laminectomy for cyst exposure, and invasiveness may be increased in long-segment cysts. Therefore, neuroendoscopy and intraoperative ultrasound may be more suitable for short-span cysts. Mishra et al. [41] reported that intraoperative MRI is more suitable for patients without a lumbar puncture space to perform myelography for cysts involving the sacral level.

## Features of dural defect location

**Fig. 7:**
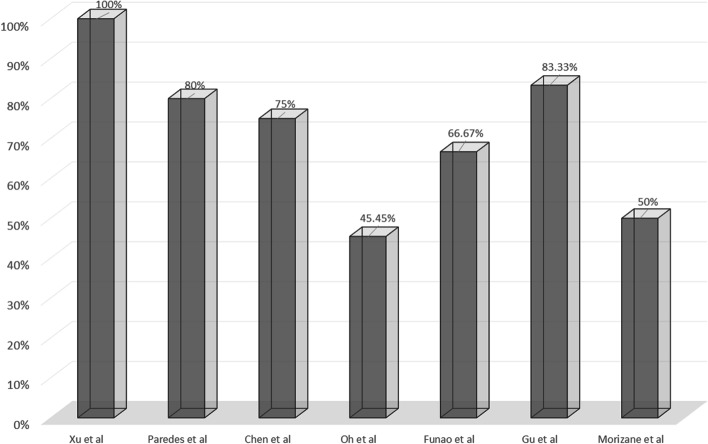
The rate of dural defects at T12 or L1 levels in the different series

Lee et al. [13] summarized the location of 51 SEMCs and proposed that cysts occur most frequently at the T12–L1 level, with a gradual decrease cranially and caudally. If there is a characteristic distribution of the location of the clefts, like SEMCs, then an empirical exploration can be effective. We searched PubMed for English articles published over the last 10 years (May 2011~May 2021). The search terms were “epidural arachnoid cyst,” “dural cyst,” and “epidural meningeal cyst.” Case reports, studies with a sample size less than four, controversial ventral dural dissection(s), and undocumented fistula locations were excluded. Seven articles were finally included (Fig. 7).

Funao et al. [12] summarized the distribution of fistulas in 12 patients with SEMC and found that most fistulas were located at the T12–L1 segment (8/12) and in the middle segment of the cyst. Therefore, when a fistula was not detected preoperatively, they recommended limited laminectomy at the middle segment of the cyst or at the T12–L1 segment and microscopic suture after detection with neuroendoscopy. Xu et al. [17] summarized the data of ten patients with thoracolumbar SEMCs and proposed that the fistula was located at the T12–L1 segment (10/10) near the nerve root. They suggested that hemilaminectomy or laminectomy be performed at the T12–L1 segment to explore and suture the fistula.

Some scholars did not directly propose the characteristics of fistula location, but we made a summary according to the fistula locations reported by them. Gu et al. [29] reported six cases of SEMC, and in five of the cases, the fistula segment was located at T12–L1. Morizane et al. [24] summarized the characteristics of 12 SEMC cases and found that 11 cysts (11/12) had fistulas, of which six fistulas were located at the T12–L1 segment and one patient had three fistulas located at T7. The fistula was also found to be located at the point of diffuse idiopathic osteoplasia, wedged vertebra, and disc herniation. They believed that the cyst was secondary to mechanical stress. Chen et al. [4] reported the location of fistulas in four cases of SEMC cysts, of which three were at the T12–L1 segment and one at T11. Oh et al. [42] reported 14 cases of SEMC, and fistulas were found in 13 of the cases. They listed the location of the fistula in ten patients, including T12–L1 and T11 in five cases each. Paredes et al. [20] reported five cases of SEMC, of which four cases had fistulas located at T12–L1, with one at L5.

In the studies reported to date, the thoracolumbar spine is the most common location of SEMCs [13]; it is a transition area between the kyphotic thoracic spine with less mobility and the lordotic lumbar spine with greater mobility. The relative overactivity of this area due to anatomical factors may induce abnormal mechanical stress, making the dura in this area more susceptible to injury, leading to the formation of SEMCs. The distribution characteristics of the fistula are particularly important for thoracolumbar cysts in which the fistula location is not identified preoperatively. Selective laminectomy or hemilaminectomy at the T12–L1 level has a high probability of identifying a fistula (Fig. 7), which can significantly reduce surgical invasion and prevent kyphosis [12].

## Conclusions and future direction

Because SEMC is very rare, a large sample-size study is difficult to conduct. Preoperative identification of the fistula location is the basis for reducing injury during spinal surgery and for preventing recurrence. At present, most studies on the examination methods for locating fistulas are case reports. The trend from static examinations in the past to dynamic examinations at present shows that examination techniques have gradually improved, but there is still no recognized gold standard or even a reasonable preferred examination. While some researchers have reported individual successes with a particular method of examination, others have reported the failures of such examinations. There is no question that a combination of various preoperative examinations, as well as intraoperative detection methods, is conducive to locate the cleft and reduce injury during operation, with several successful cases reported [18, 30]. This study summarizes the methods of locating the cleft. Future research should focus on multicenter clinical trials that aim to reveal the nature of SEMCs, improve the rate of fistula localization, and better guide minimally invasive treatment.

## Data Availability

Data sharing is not applicable to this article as no datasets were generated or analyzed during the current study.

## Abbreviations

SEMCs: Spinal extradural meningeal cysts
CSF: Cerebrospinal fluid
CT: Computed tomography
MRI: Magnetic resonance imaging
CISS: Construction interference sequence
3D: Three dimensional
T-SLIP: Time-spatial labeling inversion pulse
